# Design of a Multiparametric Perfusion Bioreactor System for Evaluating Sub-Normothermic Preservation of Rat Abdominal Wall Vascularized Composite Allografts

**DOI:** 10.3390/bioengineering11040307

**Published:** 2024-03-25

**Authors:** Sara Salehi, Ernesto Lippert Lozano, Yichuan Zhang, Yinan Guo, Renee Liu, Kenny Tran, Franka Messner, Gerald Brandacher, Warren L. Grayson

**Affiliations:** 1 Translational Tissue Engineering Center, Johns Hopkins University School of Medicine, 400 N. Broadway, Smith 5023, Baltimore, MD 21231, USA; ssalehi4@jhmi.edu (S.S.); elipper1@jhu.edu (E.L.L.); rliu23@meei.harvard.edu (R.L.); kenny.tran81794@gmail.com (K.T.); 2 Department of Biomedical Engineering, Johns Hopkins University School of Medicine, Baltimore, MD 21218, USA; yzhan317@jhmi.edu; 3 Vascularized Composite Allotransplantation Laboratory, Department of Plastic and Reconstructive Surgery, Johns Hopkins University School of Medicine, Baltimore, MD 21287, USA; yguo55@jhmi.edu (Y.G.); franka.messner@i-med.ac.at (F.M.); gbranda2@jhmi.edu (G.B.); 4 Department of Visceral, Transplant and Thoracic Surgery, Innsbruck Medical University, 6020 Innsbruck, Austria; 5 Department of Materials Science and Engineering, Johns Hopkins University, Baltimore, MD 21218, USA; 6 Institute for Nanobiotechnology, Johns Hopkins University, Baltimore, MD 21218, USA; 7 Department of Chemical and Biomolecular Engineering, Johns Hopkins University, Baltimore, MD 2121, USA

**Keywords:** vascularized composite allotransplantation, machine perfusion, bioreactors, sub-normothermic preservation

## Abstract

Static cold storage (SCS), the current clinical gold standard for organ preservation, provides surgeons with a limited window of time between procurement and transplantation. In vascularized composite allotransplantation (VCA), this time limitation prevents many viable allografts from being designated to the best-matched recipients. Machine perfusion (MP) systems hold significant promise for extending and improving organ preservation. Most of the prior MP systems for VCA have been built and tested for large animal models. However, small animal models are beneficial for high-throughput biomolecular investigations. This study describes the design and development of a multiparametric bioreactor with a circuit customized to perfuse rat abdominal wall VCAs. To demonstrate its concept and functionality, this bioreactor system was employed in a small-scale demonstrative study in which biomolecular metrics pertaining to graft viability were evaluated non-invasively and in real time. We additionally report a low incidence of cell death from ischemic necrosis as well as minimal interstitial edema in machine perfused grafts. After up to 12 h of continuous perfusion, grafts were shown to survive transplantation and reperfusion, successfully integrating with recipient tissues and vasculature. Our multiparametric bioreactor system for rat abdominal wall VCA provides an advanced framework to test novel techniques to enhance normothermic and sub-normothermic VCA preservations in small animal models.

## 1. Introduction

Vascularized composite allotransplantation (VCA) has emerged as a viable treatment option for patients suffering from the loss of myocutaneous or osteomyocutaneous tissue structures such as the face, arms [1], abdominal wall [2], and genitalia [3]. Since the first hand transplantation in 1998, more than 200 vascularized composite allotransplantations have been performed [4]. Despite promising outcomes, vascularized composite allotransplantation has not become a clinical standard; the logistics of VCA procurement, allocation, and preservation are complex, and surgeons have a short window of time to coordinate the transplantation procedure [5]. A major reason for this limited time frame is that static cold storage (SCS), the clinical gold standard, can only preserve composite tissue allografts for up to 6 h due to their highly ischemia-sensitive muscular components [6]. Efficient organ preservation is crucial for donor organ quality and viability, which impact the likelihood of graft survival. SCS provides a simple and effective way to preserve and transport organs. However, this method is insufficient in maintaining solid organs with extended preservation criteria as well as complex grafts, such as VCA, for extended periods of time.

Decades of research focused on extending SCS preservation time, often by modifying preservation solutions and cooling protocols, have resulted in limited improvements. Machine perfusion (MP) has recently gained interest as an alternative to SCS preservation [7]. In contrast to SCS, where tissues are preserved by suppressing cellular metabolism at low temperatures [8], MP aims to maintain metabolism—often at near-physiologic levels—by providing sufficient nutrients and decreasing the buildup of toxic by-products. Randomized clinical trials have shown MP to be safe and effective in improving a transplant’s short-term outcome in the heart [9] and liver [10], but protocols are still being developed for VCA. 

Most of the studies on VCA MP have been focused on demonstrating its feasibility. Many researchers have used commercial perfusion instruments developed for kidney/liver transplants or heart–lung machines with blood-based solutions as perfusates. In 2016, Ozer et al. [11] perfused swine limbs using a commercial perfusion circuit [12] with diluted blood. Moreover, they perfused a human arm with plasma and packed red blood cells for 24 h while evaluating hemodynamic parameters. Acellular perfusates and hypothermic MP have also been investigated: Krezdorn et al. [13] conducted hypothermic MP of pig forelimbs with oxygenated modified Steen solution for 24 h. The grafts preserved by MP exhibited better survival after re-implantation compared to those preserved by SCS. Thus far, the reported data suggest that extending VCA preservation time by optimizing MP protocols is feasible. However, determination of the optimal parameters remains a major challenge. 

Variations in tested MP protocols and VCA anatomies complicate the development of a standard and optimized system. A limited number of studies have investigated the impact of a few perfusion parameters. Using pig forelimbs, Amin et al. [14] varied MP pressure (70 vs. 30 mmHg) and temperature (normothermia vs. hypothermia) over 6 h after the grafts had already been preserved for 2 h by SCS. They found that both the higher temperature and perfusion pressure reduced perfusion-induced damage. Vascular resistance was also studied by Fahradyan et al. [15] as a predictive parameter for MP endpoint. They perfused five porcine forelimbs until the vascular resistance rose in comparison with the 12 h perfused graft. No statistically significant difference was observed between the two groups. Hence, it was concluded that vascular resistance is a reliable parameter to suggest cessation of MP. Beyond these and similar studies, limited information has been reported on the correlation between MP parameters and VCA viability. Hence, this dearth of information hinders the clinical translation of MP systems for VCA. 

A major challenge in advancing VCA perfusion toward clinical translation is the cost of research. VCA perfusion studies are most often conducted with large animal models, which tend to have limited sample sizes due to their cost. In contrast, the lower expenses and shorter life cycles associated with small animal models allow for more comprehensive transplantation studies with larger sample sizes. Additionally, there are many genetically defined and well-characterized rodent strains for research on transplantation and immune modulation [16]. High-throughput screening and a wide range of biomolecular tests designed for rodents also facilitate essential mechanistic studies. Here, we describe the development of a multiparametric bioreactor MP system for rat abdominal wall preservation. We demonstrate the use of our bioreactor system in a small-scale transplantation study, in which the perfusion, transplantation, and post-operative survival of rat abdominal wall VCAs are evaluated. The results reported here will inform further studies in developing and improving VCA MP systems for small animal models. 

## 2. Materials and Methods

All animal usage and experiments were conducted with National Institutes of Health guidelines and were approved by the institutional Animal Care and Use Committee at Johns Hopkins University School of Medicine. For this study, 8–10 weeks old male Lewis rats were purchased from Charles River. Rats on average weighed 350 g in the SCS-transplantation study, 400 g in the MP-only study, and 300 g in the MP-transplantation study. 

### 2.1. VCA Procurement Surgery

The abdominal wall grafts were procured based on the procedure previously described by Broyles [17]. Full-thickness, hemi-abdominal wall flaps included skin, subcutaneous tissue, fascia, and underlying abdominal muscles. Rats were put into an induction chamber to be anesthetized with 2% isoflurane for 15 min. To assure the consistency of the graft size, the area of the grafts was demarcated on each animal, which was bound from linea alba to the mid-axillary line laterally and from inferior rib cage to inguinal ligament. Rats were placed in a supine position and a 1–2 cm incision was made along the inguinal ligament lines. Superficial inferior epigastric vessels (from femoral vessels) and the proximal part of the femoral vessels were exposed and skeletonized. An incision of graft skin and muscle was made along the pre-marked lines, and the margin of muscle was cauterized afterward. The flap was then averted to access the external iliac vessels, where all their branches were carefully exposed and ligated except for the inferior epigastric vessels. All the remaining connective tissue was removed at the end to lift the flap. After weighing the flaps (W1), a blunt flushing needle (22 G) and a catheter (21 G) were inserted into the external iliac artery and vein. Blood was flushed out by injecting 4 mL of heparinized solution (perfusate or preservation solutions with heparin 250 U/mL). 

### 2.2. Cold Storage

Six grafts were procured from three donor rats. They were flushed with Perfadex, which was supplemented with heparin (250 U/mL). After blood removal, they were wrapped with Perfadex-soaked gauze and placed in plastic bags. Throughout the preservation time, grafts were stored at 4 °C. There were three preservation times: 12 h, 2 h, and 48 h (n = 2 per group) labeled SCS-12, SCS-24, and SCS-48, respectively. Syngeneic transplantations were conducted with grafts from the SCS-24 and SCS-48 groups to evaluate ischemic injury and graft survival. Muscle biopsies taken from the muscle tissue right before the transplantation procedure were marked as post-operative day 0 (POD0). 

### 2.3. Transplantation

**Figure 1:**
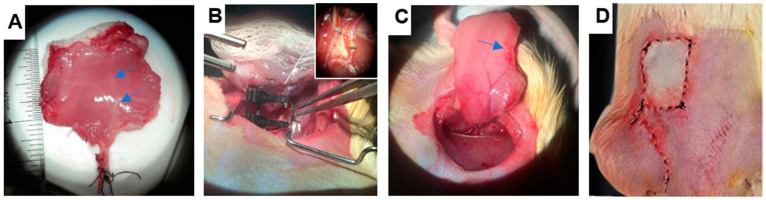
Abdominal wall syngeneic transplantation with anastomosis of the donor’s external iliac and recipient’s femoral vessels. (**A**) An abdominal wall graft after blood removal (blue arrows show flushed deep inferior epigastric artery). (**B**) Connecting donor external iliac to recipient femoral artery (cuff technique). (**C**) Blood reperfusion as a sign of successful vessels’ anastomosis (blue arrow). (**D**) Recipient rat post-transplantation. Two incisions were made: one for vessel anastomosis and one for suturing the abdominal wall graft into the recipient.

Recipients were prepared similarly to donors, and their femoral vessels were connected to donors’ external iliac vessels using the cuff technique described previously^45^. The abdominal wall graft was placed and sutured in an orthotropic position (Figure 1D). Post-transplantation muscle biopsies were taken from the superior edge of the grafts to avoid damaging the graft perfusion.

### 2.4. Machine Perfusion System

The grafts used in the MP experiments were flushed with the perfusate supplemented with heparin (250 U/mL). They were then transferred into our custom-designed bioreactor. It was iteratively designed to satisfy the design criteria defined for the abdominal wall graft perfusion. We constructed 3D printed prototypes of the bioreactor using a LulzBot Taz 5 3D printer and white acrylonitrile–butadiene–styrene (ABS) to test each design. The bioreactor was manufactured by Johns Hopkins University’s Whiting School of Engineering’s Machine Shop. It was fabricated from clear polycarbonate, a light and durable plastic material that is easy to machine. The respective length, widths, and heights of the bioreactor were 25, 14.6, and 17 cm. Stainless steel screws (#0-80 × ¾”) held the bioreactor pieces together. Before each MP experiment, the all pieces were cleaned and sterilized.

In the bioreactor, the abdominal wall graft was housed in a clean and humid environment while connected to the perfusion circuit. Perfusate drained freely out of the grafts’ periphery to a collecting reservoir which was in connection with a peristaltic pump. In addition to the bioreactor, our perfusion circuit included two peristaltic pumps, an oxygenator, a wire heater, and a real-time monitoring system. One pump (Peristaltic pump IPC-8, Ismatec, Glattbrugg, Switzerland) recirculated perfusate between the reservoir and grafts and the other one (Analog Variable-Speed Console Drive Systems, Avantor, Radnor, PA, USA) recirculated perfusate between the reservoir and oxygenator (Figure 5). The oxygenator was a 3M™ Liqui-Cel™ MM-0.5×1 Series membrane contactor with an acceptable maximum pressure of 45 psi on the liquid side and 15 psi on the gas side. The Ismatec pump had an analog interface which allowed for electronic control. 

We used Pharmed tubing (PharMed® BPT Biocompatible, ColeParmer, Vernon Hills, IL, USA) with an oxygen permeability of 80 × 10^−10^ $\frac{cc-mm}{sec-{\mathrm{cm}}^{2}-cmHg}$. The pressure against perfusion into the inferior epigastric artery was measured by using an 8-730 pressure transducer from AD Instrument Inc. Perfusate pH, dissolved oxygen, and temperature were also measured by using microelectrodes (8-702, 8-705, A475, AD Instrument Inc., Colorado Springs, CO, USA) installed in the perfusate reservoir. The perfusate temperature was increased to 22–24 °C with the wire heater wrapped around the perfusate reservoir. The wire heater was controlled by a separate external circuit. The heater was capable of increasing perfusate temperature and maintaining it in the range of 35–37 °C. However, we conducted sub-normothermic MP due to limitations in oxygen delivery. All the sensors were connected to the 16/35 PowerLab (AD Instruments, Colorado Springs, CO, USA). LabChart software (Version 8, AD Instruments, Colorado Springs, CO, USA)was used as a data acquisition and analysis platform for all the recording sensors. In the case of perfusate leakage from the intubated vessels, the MP experiments were terminated, and their associated data were excluded from the dataset. 

The bioreactor was equipped with an electrical stimulation system as a method of viability assessment. The electrical stimulation system comprised of a signal generator (Powerlab stimulator outlet), connecting wires, electrodes, and a force sensor. Trains of stimulus could be adjusted as needed. The stimulation frequency could be varied from a single pulse up to 150 Hz with voltage ranges from 0 to 5 V. A force transducer (MLT1030/D by ADInstruments, Colorado Springs, CO, USA) with a measurement range of 0–200 g was used. It was attached to the back wall of the bioreactor by a screw to make its position adjustable. Self-adhesive surface electrodes (reusable TENS skin electrodes) covering the muscle surface were found to be sufficiently reliable and stable in stimulating the muscle tissue. A set of tissue clamps inside of the bioreactor were utilized to hold one side of the graft in place and record contraction force by measuring the blade deflection of the force transducer. The transducer had 10 blades which expanded its measurement range, and just one blade was sufficient to cover the graft range of contraction force (0–25 g). It was connected to an external laptop with LabChart 8 (AD Instruments, Colorado Springs, CO, USA), and contraction force and other sensors readouts were recorded in real-time.

Within two days before every perfusion experiment, each perfusion circuit device was cleaned and perfusate was prepared. Perfusate components such as dexamethasone and insulin were added to the perfusate right before beginning the perfusion. On the day of the experiment, the system was assembled and primed with lactated Ringer’s solution. The perfusate outflow reservoir was sampled every two hours, and perfusate content was evaluated with a blood gas analyzer (ePOC, Heska, Loveland, CO, USA). At the end of the experiment, the grafts were weighed and sampled prior to transplantation. Vascular resistance was calculated through Ohm’s law applied to hemodynamics, R = (arterial pressure − venous pressure)/(flow rate); here, arterial pressure was measured by the inline pressure sensor and venous pressure was 0 due to open outflow.

### 2.5. Histology

Muscle samples were dipped in OCT, snap-frozen in iced isopentane, and stored in a −80 °C freezer for later processing. Cryopreserved samples were cut into 10 µm thick sections, collected with Superfrost Plus slides, and air-dried overnight. 

One slide per sample (6 slides per graft) was stained with hematoxylin and eosin (H&E) to evaluate necrosis and perfusion-induced damage. Random regions on the slide were picked to be imaged on a Zeiss Axio Observer 7 (Zeiss, Pleasanton, CA, USA)with a 20 × objective (6 images per slide). The percentage of myofiber cross-sectional area showing prominent necrotic attributes, including non-homogenous eosinophilic staining, hypercontraction, vacuole formation, and infiltration, was measured in every image. The average percentage was used to assign a necrosis score to each sample. Each graft necrosis score is an average score of its six samples. Usually, disruption of epimysium and separated myofiber are considered to be signs of interstitial edema. Because of the high inter-observer variability, we quantified edema as a percentage of white area in between myofibers in images taken from H&E-stained samples. The original images were converted to binary, and the percentage of white pixels was recorded. For each sample, the average value obtained from six images was normalized to the value of pre-ischemic samples obtained in our preliminary study. 

To assess and quantify necrosis, a TUNEL assay was performed on the cryopreserved muscle samples with a TACS 2 TdT-DAB In Situ Apoptosis Detection Kit (R&D Systems). Following rehydration and fixation, one slide per sample was covered with cytonin at room temperature for two hours. A TdT-based labeling reaction mix was formulated and added to each slide before incubating it for one hour at 37 °C in a humidity chamber. The slides were incubated for an additional 10 min with streptavidin–horseradish peroxidase, the activity of which was indicated by oxidation of the added diaminobenzidine. Slides were counterstained with 5% methyl green. The quantification of necrotic nuclear fraction was calculated by first filtering stained and counterstained areas by color threshold and size, and then dividing the number of positively stained nuclei by the number of total nuclei in each image.

### 2.6. Statistical Analysis

All values were reported as a mean with a standard deviation of three grafts (n = 3). Statistical analysis was performed using GraphPad Prism 5 software. Pressure, pH, and oxygen readings are presented by line graphs, and their mean values with standard deviation at two-hour time intervals are marked on the graphs. Statistical significance was determined by *t*-test or one-way ANOVA. 

Additionally, 2% Evans blue dye (Sigma-Aldrich, Burlington, MA, USA, E2129-10G) was prepared in saline. The dye was used as the initial test for peripheral perfusion within the abdominal wall graft at different time points of MP. Images were taken by camera from the surface of the muscle. Stained muscle provided evidence for peripheral perfusion.

## 3. Results

Our study was divided into two modules. In the first module, we developed a multiparametric bioreactor integrated with a perfusion circuit and non-invasive sensors. This system was used to establish a perfusion protocol that minimized damage to the rat abdominal wall VCA. In the second module, we evaluated the ability of the bioreactor system to maintain graft health over several hours. We assessed this through the collection of physical and biomolecular metrics and further validated them in transplantation procedures and histological assessments.

### 3.1. Quantitative Metrics of Necrosis Were Established to Study the Correlation between Ischemic Damage and Graft Survival in a Transplantation Model of Rat Abdominal Wall VCA Grafts

Syngeneic transplantation provides an ideal platform to study ischemia-reperfusion injuries (IRI) [18]. We validated the previously established model of syngeneic rat abdominal wall transplantation (Figure 1) for investigating ischemic injury during preservation. Throughout the preservation–transplantation procedure, grafts were subjected to both warm and hypothermic ischemia conditions. The average duration of the donor procedure (graft harvest, vessel preparation, and flushing) prior to graft transfer to cold storage was roughly 60 min. The grafts were randomly assigned to three groups of SCS preservation times: 12, 24, and 48 h. To further study the impact of SCS preservation, VCAs preserved for 24 and 48 h were transplanted to recipients following cold storage. 

Muscle is highly vulnerable to ischemia in VCA grafts due to its high metabolic rate [19]. Metabolic failure leads to a series of observable events, the first stages of which are indicated by pale cytoplasm and ruptured plasma membranes in H&E-stained myofibers. Later stages involve phagocytosis of the necrotic myofibers and collapse of the affected regions [20]. To quantify necrosis without interobserver variability, a necrosis score was assigned to muscle samples based on the percentage of myofibers presenting any of the ischemic signs. In addition to myofiber necrosis, fiber separation associated with extracellular edema was quantified by assessment of the white space in between myofibers in H&E-stained muscle samples. Histological images of the abdominal wall grafts preserved under SCS demonstrated the progression of hypothermic ischemic damage over time (Supplementary Figure S1A). Within 12 h, hypercontraction and pale cytoplasm were present in limited regions of muscle tissue. This hypercontraction, coupled with dispersed phagocytosis, appeared in the SCS24 grafts. A significant disruption in the perimysium and collapsed regions was evident in the SCS48 grafts. Myofiber separation increased over time, mainly due to collapsed necrotic cells (Supplementary Figure S1B). Although overall weight gain remained relatively low (Supplementary Figure S1C), graft weight also increased over time.

The association between ischemic injury and transplantation outcome was studied by transplanting abdominal wall grafts preserved with 24 and 48 h SCS. The muscle remained edematous up to post-operative day 7 (POD7) (Supplementary Figure S2A–C). The skin healed fully within two weeks (data not shown), and skin and hair growth were found to be normal by POD50 (Supplementary Figure S2D). Histological assessments of post-operative graft tissue demonstrated the localized infiltration of mononuclear cells within the first week (Supplementary Figure S2F,G). At POD50, the myofibers appeared significantly smaller in diameter, suggesting some atrophy (Supplementary Figure S2H). The grafts preserved for 48 h exhibited pale skin upon transplantation, and necrotic areas became visible on the skin within one day (Supplementary Figure S2I,J). The recipients were subsequently euthanized. Corresponding necrosis in the histological assessments from samples harvested before and after transplantation was also observed (Supplementary Figure S2K,L). The quantitative necrosis data suggest that grafts preserved for 48 h exhibited significantly higher necrosis at POD0 than grafts preserved for 24 h, though similar levels of myofiber separation were observed between the two groups (Supplementary Figure S2M–P). 

### 3.2. Custom-Designed Bioreactor Housing Unit for Rat Abdominal Wall VCA Grafts

**Figure 2:**
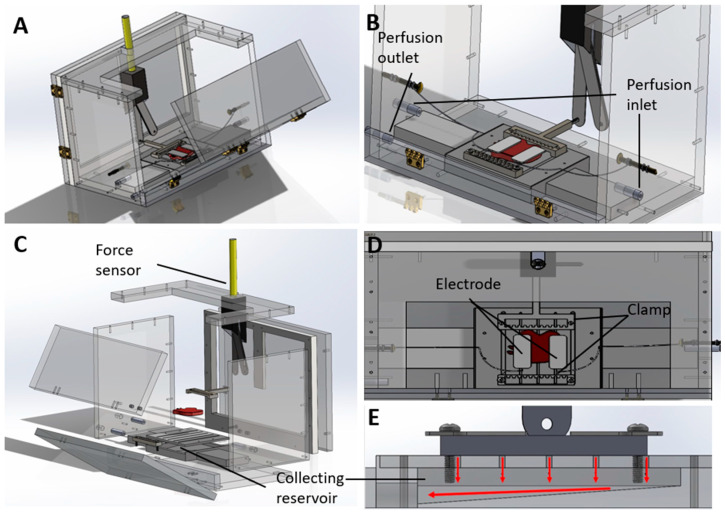
Bioreactor housing designed for the rat abdominal wall VCA. (**A**–**E**) CAD drawings from different views showing the bioreactor’s main parts including force sensor, electrodes, perfusion inlet and outlet, tissue clamp, and collecting reservoir.

The graft housing unit was designed iteratively to satisfy key design criteria, including minimization of contamination risk, maximization of graft stability, access to perfusion ports, and facilitation of adequate drainage (Figure 2) The hemi-abdominal wall VCA graft was comprised of several muscle layers, including the transversus abdominis, internal and external obliques, and a thin layer of rectus abdominis [21]. The inferior epigastric artery was connected to the perfusion loop to deliver nutrients to the muscle tissues. However, given the anatomy of graft, the vasculature did not form a closed loop with a single inlet and outlet. Cauterizing the edges of muscle tissue to enclose the vascular structure results in tissue swelling; therefore, the edges were cut to allow perfusate to drain freely from the periphery of the graft. Consequently, our bioreactor design was unable to obtain inline oxygen measurements from the perfusate outflow–a common metric evaluated in other machine perfusion studies [22]. Instead, the bioreactor was designed to contain a drainage compartment from which perfusate outflow could be collected for recirculation and analysis. Clamps were designed to provide a stable connection between the graft and the intubating needle to minimize damage to the tissue. The doors and side walls of the bioreactor were designed to provide easy access to the graft throughout the perfusion. 

The bioreactor housing unit included two sliding panels at the back and a large folding front panel (Figure 2A) to facilitate easy placement and access to the grafts as needed. The facile assembly and disassembly protocols allowed for the reusable bioreactor units to be cleaned and sterilized prior to use. The assembled and exploded views are shown in Figure 2B,C. All panels remained closed during perfusion to maintain humidity and minimize contamination during perfusion. The abdominal wall grafts sat on a grated base (Figure 2D) that allowed the perfusate to drain into a reservoir below (Figure 2E). The collecting reservoir was connected to the pump to recirculate the perfusate. The connecting ports placed on the two side walls allowed for simultaneous preservation of both left and right abdominal wall grafts. Once placed on the base with the ventral surface (skin side) facing down, the graft was sutured on both sides (along the linea alba and the midaxillary line) to clamps which hold the graft securely in place during perfusion. Clamping the grafts also facilitates the placement of electrodes (Figure 2B,D) for electrical stimulation and contractile force measurement. Interrupted sutures are used to secure the grafts to the clamp so as not to obstruct the outflow of perfusate from the graft periphery. One clamp can be connected to a force sensor. Another is attached to the graft base and can be adjusted by sliding through the slits of the base such that the graft is placed initially at its resting length. The walls of the bioreactor are transparent so that the graft can be visibly observed throughout the preservation phase.

### 3.3. Electrical Stimulation of Rat Abdominal Wall VCA Grafts

Our electrical stimulation was designed to effectively stimulate muscle tissue in the abdominal wall grafts during MP and measure the force of contraction to evaluate ischemic damage. To uniformly stimulate the denervated muscle, we implemented an electrical field stimulation system in the bioreactor. An isolated signal generator was connected to two copper connections in the bioreactor side walls. The abdominal wall muscle layers had varied myofiber alignment which was disconnected from their motor points; hence, a preliminary study was conducted to find the direction of the graft contraction. The transversus abdominis was found to be dominant in producing contraction forces, measured by a force sensor. 

In electrical field stimulation of denervated muscle, electrodes were required to stimulate the muscle directly. Wire electrodes made of platinum and copper that were placed with electrolyte gel failed to stimulate the muscle tissue uniformly. Surface self-adhesive electrodes were found to be consistent and stable (Supplementary Figure S4A). They were used to transfer biphasic 1 Hz step signals. Since the electrical stimulation system was designed to evaluate the muscle during MP, the signal amplitude remained consistent. MP did not prolong the contraction force decline over time, potentially due to the lack of oxygen carriers in the perfusate that would support the higher metabolic rates needed during contraction (Supplementary Figure S4B). 

### 3.4. Machine Perfusion Circuit

**Figure 3:**
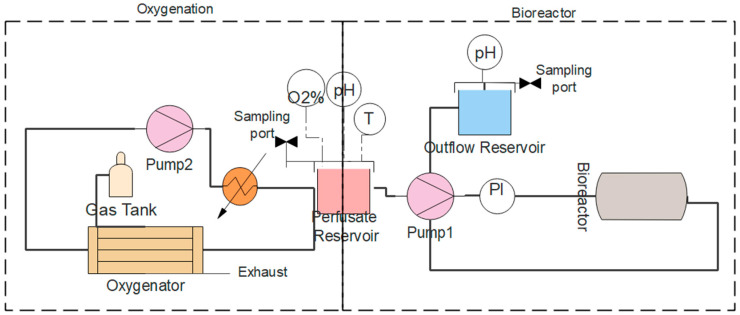
The MP circuit to perfuse oxygenated sub-normothermic perfusate into the rat abdominal wall VCA graft. The perfusion circuit consisted of two pumping circuits, an oxygenator, and a bioreactor; the oxygenator pump recirculated perfusate through the oxygenator and perfusate reservoir, and the bioreactor pump transferred oxygenated perfusate from the perfusate reservoir to the bioreactor. Image of the MP system is presented in Supplementary Figure S7.

The perfusion loop through the bioreactor was comprised of the perfusate reservoir, a pump, an inline pressure sensor, and the outflow reservoir (Figure 3). Up to four sensors could be connected to the data acquisition unit for real-time monitoring. To decrease the required volume of perfusate, we minimized the length of tubing and the number of in-line devices. Oxygen, temperature, and pH sensors could be installed in the perfusate reservoir. Additionally, heated wire could be wrapped around the reservoir for temperature control. In the initial testing, the flow rates of perfusate into the abdominal wall graft ranged from 0.1 to 0.5 mL/min. This flow rate was too low for use with the hollow fiber membrane oxygenator, which required 5–50 mL/min for surface area of 0.1 m^2^. Therefore, we implemented a second perfusion loop to oxygenate the perfusate (Figure 3) that operated using higher flow rates (5–20 mL/min). Consequently, the perfusate reservoir also served as an intrinsic bubble trap. Since the perfusate was recirculated throughout the perfusion circuit, we can assume the fluid was sufficiently mixed and the main mass transfer resistance was in the gas–liquid layer. We experimentally determined the volumetric mass transfer coefficient in the membrane contactors at different gas pressures and fluid flow rates (Supplementary Figure S5). A gas pressure of 300 mmHg (99% oxygen) and a minimum flow rate of 10 mL/min were found to support the highest mass transfer in our system. 

### 3.5. Pressure-Controlled Perfusion and Modified Perfadex Composition Minimized Perfusion-Induced Damage in the Abdominal Wall VCA

HTK (histidine-tryptophan-ketoglutarate) and Perfadex, two common preservation solutions, were evaluated as baseline perfusate fluids, and albumin and dextran were evaluated as supplementary colloids. Perfusing the abdominal wall flap with Perfadex with added dextran (Perfadex-Dex) yielded a weight gain of 20.2 ± 7.5% and slightly lower perfusion pressure compared to the other experimental groups (Supplementary Figure S6A–D). The weight gain obtained with Perfadex-Dex was promising in comparison with several weight gain values (26–58%) reported in previous VCA MP studies [23]. Thus, we decided to use Perfadex-Dex in the following MP experiments.

While using Perfadex-Dex, the pH of the perfusate dropped to ~5 within the first 2–4 h of the abdominal wall graft MP. Decreasing pH is known to be a sign of anaerobic metabolism, acidosis, and ischemic injury [24]. Through titration studies, we found that providing an additional 8 mM THAM stabilized pH during MP without affecting weight gain or leading to higher perfusion pressures (Supplementary Figure S6E–G). To determine whether the initial high perfusion pressures were leading to increased swelling, we tested two perfusion regimens with either constant flowrate (0.2 mL/min) or constant pressure (40 mmHg—half of the femoral artery systolic pressure [25]) with the optimum perfusate described above. We found that perfusing at constant pressure resulted in decreased tissue weight gain (Supplementary Figure S6H,I).

### 3.6. Evaluation of Physical, Biomolecular, and Histological Metrics during and after Perfusion of Rat Abdominal Wall Grafts

After optimizing the parameters pertaining to bioreactor circuit design and perfusate composition, a small perfusion and transplantation study was designed to evaluate the system’s ability to support abdominal wall VCA viability and report on physical and biomolecular metrics throughout the course of extended perfusion at room temperature. The bioreactor circuit design was adjusted for the following experiment. In particular, components of the circuit related to temperature control and oxygenation were removed to reduce the variable elements of the study. Nine abdominal wall grafts were harvested from the donor Lewis rats and assigned to 2, 6, or 12 h of perfusion (n = 3 per group). 

**Figure 4:**
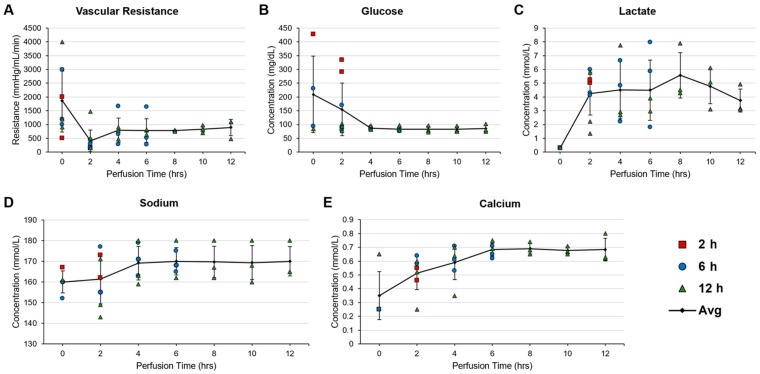
Trends in metrics collected throughout the duration of abdominal wall machine perfusion from inline sensors and analysis of perfusate outflow. (**A**) Vascular resistance remains stable over 12 h of perfusion. (**B**) Concentration of glucose in the perfusate outflow indicates steady rate of uptake by the graft. (**C**) Presence of lactate in the perfusate outflow over time is variable but stable. (**D**,**E**) Stability of electrolytes in perfusate outflow suggests consistent cell membrane integrity over time.

We aimed to evaluate graft viability during perfusion through non-invasive and real-time data collection of informative and obtainable metrics. Five such metrics were selected to represent aspects of graft health: vascular resistance as a measure of vascular integrity; glucose concentration in perfusate (as a proxy of cellular glucose uptake); lactate concentration in the perfusate (as a measure of cellular metabolic activity); and sodium and calcium concentrations in the perfusate to assess cell membrane stability and ion leakage [14,15,22]. The resulting values are reported in Figure 4. Vascular resistance was found to remain consistently stable throughout 12 h of perfusion following initial fluctuations (Figure 4A). Similar results were observed for glucose, where low, stable values were indicative of consistent cellular uptake (Figure 4B). Lactate concentration in the perfusate outflow was more variable, with initial increases were attributed to anaerobic respiration stabilizing over time (Figure 4C). Cellular membrane stability as reported by sodium and calcium concentrations was impacted by the unoxygenated environment, as indicated by the initial increases in extracellular electrolyte concentration (Figure 4D,E). The concentrations of these electrolytes stabilized over the course of the longer perfusions.

**Figure 5:**
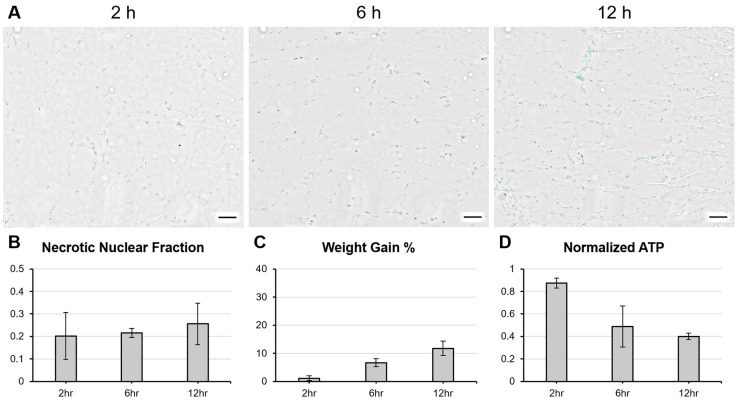
Assessments of cellular necrosis, ATP depletion, and weight gain in abdominal wall grafts following 2, 6, and 12 h of machine perfusion. (**A**,**B**) Proportion of nuclei positively stained by TUNEL (methyl green counterstain) slightly increases with perfusion time. Scale bars: 200 µm. (**C**) Normalized intracellular ATP decreases over longer perfusion times. (**D**) Graft weight gain increases over longer perfusion times, not exceeding 15%.

Upon cessation of each perfusion, muscle samples were taken for further analysis and grafts were weighed. Harvested muscle was used for the quantification of intracellular ATP, an early indicator of ischemic injury progression [22]. Terminal deoxynucleotidyl transferase dUTP nick end labeling (TUNEL) was additionally performed on harvested muscle samples for the detection of necrotic cell death. Typically used as a measure of apoptosis, TUNEL staining methods can also highlight cell death due to necrosis, such as that caused by ischemic injury [26]. Staining with TUNEL indicated no significant differences in cell death with longer perfusion times, with between 20 and 25% of counted nuclei stained positively in grafts perfused for up to 12 h (Figure 5A,B). Weight gain increased with longer perfusion times, but remained under 15%, implying adequate perfusate drainage and minimal interstitial edema (Figure 5C). Normalized intracellular ATP at the perfusion endpoint decreased with perfusion time (Figure 5D). 

### 3.7. Successful Transplantation and Integration of Perfused Abdominal Wall Grafts

**Figure 6:**
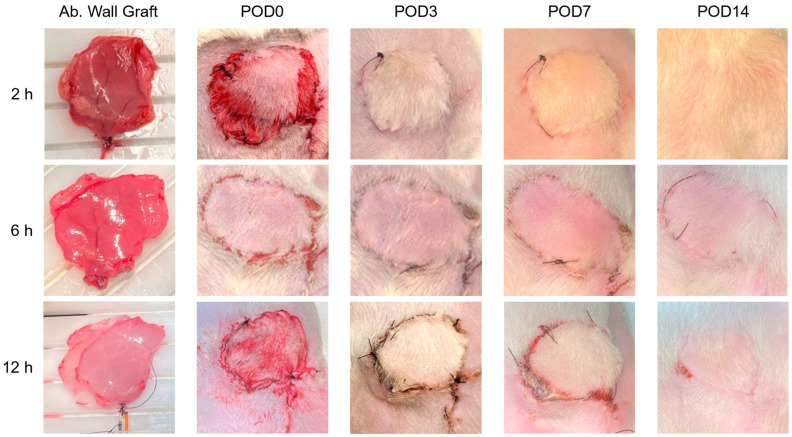
Assessment of graft integration and health during post-operative recovery period. Recipient rats were monitored for 14 days. Flushed appearance of the skin suggests successful reperfusion in grafts perfused for 2, 6, and 12 h. Signs of necrosis, marked by dark discoloration on the skin, were not observed.

Syngeneic transplantation procedures were performed immediately following the cessation of each perfusion, and recipient rats were monitored for two weeks. Images of the transplantation site were taken upon completion of the surgery, as well as 3, 7, and 14 days afterward (Figure 6). All grafts perfused for 2 and 6 h were transplanted successfully, and recipient rats recovered over the course of the monitoring period with no complications. Of the three grafts transplanted after 12 h of perfusion, two remained viable throughout the monitoring period. A third transplanted graft was not adequately re-perfused by the recipient’s vasculature due to surgical complications, and thus did not survive. Among the surviving transplanted grafts, skin appeared flush and lacked necrotic discoloration, and swelling consistently subsided over the course of the monitoring period. No other indications of IRI were detected.

## 4. Discussion

VCA grafts with multiple tissue types cannot be well preserved by SCS for extended periods of time, restricting their availability. MP has been studied as an alternative by removing metabolic toxins from the tissue while essential nutrients and therapeutic agents can be delivered. Thus, it is possible to not only extend preservation times but also improve graft viability before transplantation. MP systems have been established in the market for a few solid organs. Further, multiple research studies have revealed the feasibility of MP in VCA preservation by demonstrating stable perfusate flow dynamics with relatively low tissue swelling [11,27]. Given the variability in VCA models’ anatomy and the complexity of MP, further investigation into the MP parameters and graft viability is critical for translating such technologies. 

Typically, large animal models have been utilized in VCA MP preservation research. Although such models are anatomically relevant to humans, conducting perfusions and surgeries with them requires significant investments of time and resources. Small animal models benefit transplantation research with relatively lower cost and easier maintenance. Moreover, the well-studied transplantation rat models (allogeneic and syngeneic) in combination with genetic modification and immune modulation tools provide opportunities for both mechanistic research and therapeutic development [25]. Recently, rat hind limb VCA-MP models have been developed by Gok and Fichter [26,27]. Expanding to other VCA models, we studied abdominal wall grafts, where the vasculature does not form a closed network—similar to that of facial VCAs. We designed and optimized the platform with which abdominal wall VCA perfusions would be conducted, and we performed a demonstrative study using this platform to evaluate graft viability over multiple perfusion times.

Some characteristics of rat VCAs necessitate the modification of typical MP specifications. In large animal VCA MP systems, a single pump draws perfusate from a perfusate reservoir, passing it through an oxygenator and then into the graft [11,14,23]. In small animal VCA MP systems, commercial medical devices cannot be used due to a much lower flow rate range. Even with non-medical devices, the required flow rate might compromise the efficiency of devices such as oxygenators. Further, low-fluid flow rates with a given gas pressure could lead to the formation of bubbles in the tubing. To circumvent this limitation, we recirculated the perfusate with a separate pump through a hollow fiber membrane oxygenator and, through another circuit, connecting the perfusate reservoir to our bioreactor housing unit. Moreover, by recirculating the perfusate, the exposure time to oxygen is extended and leads to increased mass transfer [28,29]. 

Non-invasive monitoring of the VCA MP is essential to detect system failure and avoid affecting the viability of grafts. Generally, parameters related to the perfusion circuit (flow rate/pressure) and perfusate (pH, dissolved O_2_, CO_2_, electrolytes, lactate, and creatine kinase) can be monitored continuously [14,15,30]. In VCA MP, the peripheral perfusion of grafts has also been evaluated by semi-quantitative methods such as indocyanine green angiography, infrared thermography, and tissue oximetry [31]. Still, information on the link between these parameters and graft viability, which could predict graft survival, is lacking. We incorporated both invasive and non-invasive monitoring elements into our MP bioreactor system. A set of non-invasive metrics that could communicate the state of cellular metabolism, cell membrane integrity, and perfusion-induced damage were selected for evaluation in the perfusion/transplantation study. Also included in the study was a set of invasive metrics, similarly chosen to communicate metabolic state, cell viability, and tissue swelling. Further, we designed bioreactor components for delivering electrical stimulation and measuring subsequent muscle contraction forces. In a limitation of this study’s chosen graft model, we were not able to measure dissolved oxygen in the perfusate outflow and thereby quantify oxygen uptake because of the free flow from the graft’s periphery.

The built-in electrical stimulation component was installed to record contractile force during MP. Force of contraction as a viability measure was studied by Taeger et al. [32] in pig abdominis muscle MP. In the absence of nerves propagating the action potentials, electrodes can apply a sufficiently high stimulus to uniformly activate most of the muscle fibers [33]. A larger surface covered by electrodes activates more muscle tissue while decreasing current density. Diminished current density increases the chance of stimulation crossover, but in the bioreactor, this is not a major concern due to the muscle tissue being isolated [34]. Activating deeper muscle layers with surface electrodes alone is challenging, and the direction of abdominal wall muscle contraction (parallel to transversus abdominis muscle fibers) suggests that the deeper layers were not activated in our system. Increasing amplitude or pulse width is an alternative strategy to activate deeper fibers, but these options were disregarded as resultant damage could outweigh the benefits in ischemic conditions [33]. Therefore, we used two rectangular self-adhesive electrodes made of active carbon fiber to provide homogenous stimulation across the flat surface of the transversus abdominis muscle. Low-frequency stimulation has been associated with lower ATP consumption, so we tested a low-frequency (1–10 Hz) stimulus [35]. The amplitude was set to be the minimum that would elicit visible contraction after the procurement surgery. Despite the clinical relevance and simplicity of electrical stimulation, its application as a VCA viability assessment is limited. It is imperative to attain adequate levels of oxygenation prior to the application of electrical stimuli. In severely ischemic tissue, muscle contractions do not manifest, so we did not incorporate electrical stimulation and contraction force elements into the second module of this study. The timeline for assessing muscle contractility will be determined in future studies.

We optimized the perfusion protocol and perfusate composition to minimize perfusion-induced damage. Initially, HTK was used as the perfusate base along with added colloids albumin and dextran. HTK contains mannitol, an impermeant, which controls the transfer of water between extracellular and intracellular spaces. In contrast, colloids control water diffusion out of blood vessels [36]. The mannitol component of HTK was insufficient, and supplementing it with albumin decreased tissue swelling. This result agrees with the outcomes of heart perfusions with HTK in previous research [37]. However, large-molecular-weight components like albumin increase perfusate viscosity and have been implicated in organ dysfunction [38]. Dextran, in comparison with albumin, was found to be preferable since it did not noticeably increase perfusate viscosity. Dextran demonstrated superior results in lung hypothermic MP [39], but not in pig forelimb hypothermic MP [24]. We compared HTK to Perfadex with similar colloid concentrations to compare the effects of intracellular and extracellular preservation solutions. Extracellular solutions contain high sodium (100–138 mM) concentrations, while intracellular solutions contain low sodium concentrations (10–25 mM) [38]. The results from the present study suggest that the inclusion of the osmotic agent, dextran 40, in an extracellular solution (Perfadex) prevents perfusate leakage to the interstitial space and reduces tissue weight gain during perfusion. The superiority of an extracellular solution with colloid has previously been observed in heart and lung preservation [40].

Acidosis is one of the early signs of anaerobic metabolism in the absence of oxygen delivery. Recirculating acidic perfusate would exacerbate ischemic injury in VCA grafts, so high buffering capacity is desirable to maintain neutral pH throughout the MP. Perfadex contains THAM, a weak base with a pK_b_ of 5.91 (at 25 °C) that produces a closed buffer system with HCl [41]. We found that 0.4 mM additional THAM at the beginning of sub-normothermic (room temperature) MP can maintain perfusate the pH around 7.3, even without oxygen delivery and aerobic metabolism. With high peripheral perfusion and low perfusion-induced injury, our modified Perfadex with constant pressure setting has been validated as the optimum perfusion protocol for the rat abdominal wall muscle MP.

## 5. Conclusions

In the present study, we described a systematic approach toward the design and manufacture of a custom system for small animal VCA MP. These systems are aimed at removing metabolic toxins and delivering nutrients. As the foremost objective in developing VCA MP systems, we studied perfusion protocols to minimize perfusion-induced damage edema. We found that pressure-controlled MP using Perfadex supplemented with dextran and THAM was the optimal platform for conducting small animal VCA MP studies. We demonstrated through a small-scale perfusion and transplantation study that the viability of rat abdominal wall VCAs can be sustained for up to 12 h under these conditions. The abdominal wall VCA MP can be used as a platform for further investigating therapeutic delivery in an effort to mitigate ischemic reperfusion injury and improve transplantation outcomes. In following studies, the system has the potential to incorporate oxygen delivery molecules such as hemoglobin substitutes, which can potentially extend the preservation time with improved viability beyond 12 h.

## Data Availability

Raw data will be made available upon reasonable request.

## Supplementary Materials

The following supporting information can be downloaded at: https://www.mdpi.com/article/10.3390/bioengineering11040307/s1, Figure S1: Damage from hypothermic ischemia increases over time but with minimal impact on graft weight; Figure S2: Ischemic injuries from SCS longer than 24 hours lead to graft rejection; Figure S3: Intubation of deep inferior epigastric in abdominal wall graft provides viable access for MP; Figure S4: Direct electrical stimulation in the bioreactor can deliver biphasic signals and record the force of abdominal wall muscle contraction; Figure S5: Volumetric mass transfer coefficient in relation to perfusate (water) flow rate and gas (oxygen) pressure; Figure S6: Determining perfusate composition and perfusion protocol for sub-normothermic MP of rat abdominal wall grafts; Figure S7: Image of the MP system configured to preserve an abdominal wall VCA [42,43].
