# Supplementary material for: Design of a Multiparametric Perfusion Bioreactor System for Evaluating Sub-Normothermic Preservation of Rat Abdominal Wall Vascularized Composite Allografts
Source: Bioengineering (Basel). 2024 Mar 25;11(4):307. doi: 10.3390/bioengineering11040307 (PMC11047557; doi:10.3390/bioengineering11040307)
Supplement: Supplementary file 1 [file bioengineering-11-00307-s001.zip › bioengineering-2880808-supplementary.pdf]

Design of a Multiparametric Perfusion Bioreactor System for Evaluating Sub-Normothermic Preservation of Rat Abdominal Wall Vascularized Composite Allografts

Sara Salehi, Ernesto Lippert Lozano, Yichuan Zhang, Yinan Guo, Renee Liu, Kenny Tran, Franka Messner, Gerald Brandacher, Warren L. Grayson

Supplementary Figures

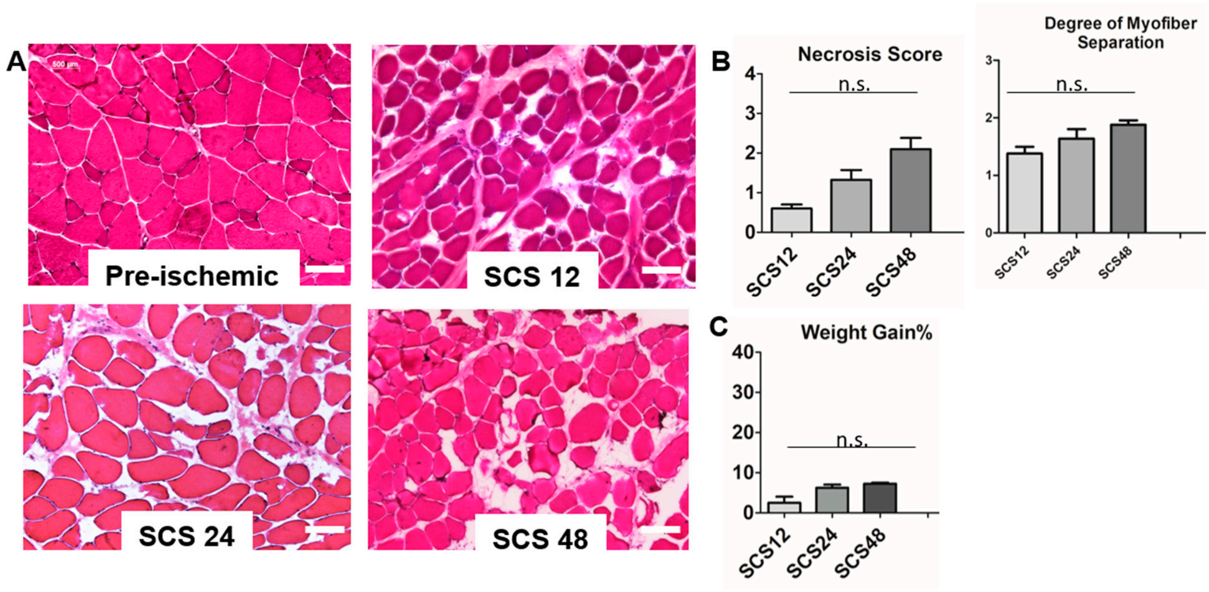

Supplementary Figure S1. Damage from hypothermic ischemia increases over time but with minimal impact on graft weight. **A.** Representative H&E images of muscle cross-sections at various static cold storage times. Scale bars: 100 µm. **B.** Morphological viability evaluation of abdominal wall muscle**. C.** Weight gain of the graft after SCS storage with Perfadex for different times. Data are shown as mean with SEM. No statistical significance.

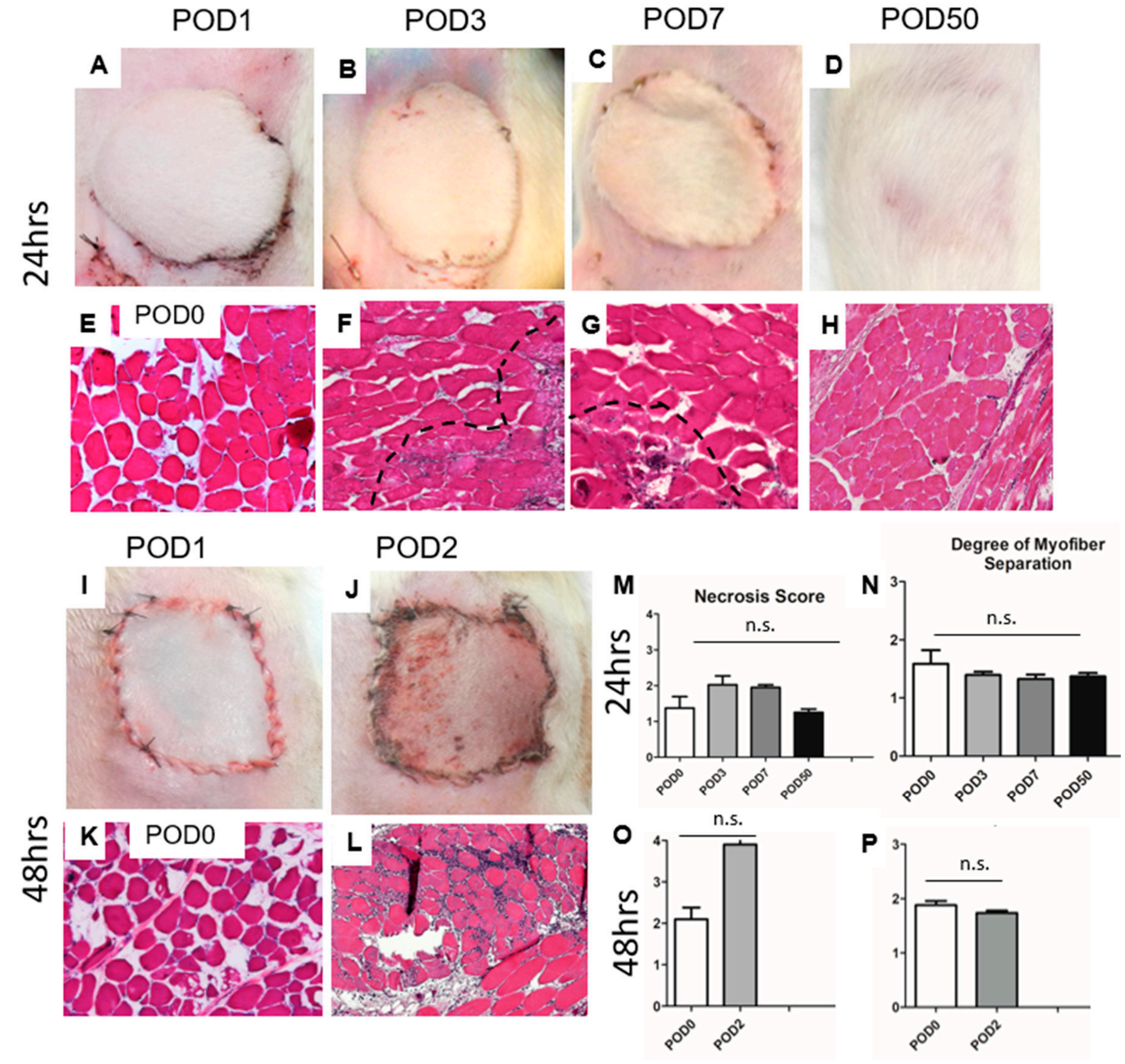

Supplementary Figure S2. Ischemic injuries from SCS longer than 24 hours lead to graft rejection. A-L. Representative macroscopic and microscopic (H&E) images of two groups (SCS24 and SCS48)of transplanted abdominal wall grafts; preserved in Perfadex cold bath for 24 (A-H) and 48 (I-L) hours. In muscle tissue from the 24-hour group, infiltration was localized and diminished over time but in the 48-hour group, infiltration impacted the whole muscle tissue. Morphological evaluation of muscle biopsy taken from the grafts before and after transplantation of 24-hour group **(M, N)** and 48-hour group **(O, P)** (1 donor and 2 recipient rats per group). Data are shown as mean +-SEM. No statistical significances were observed.

Dye perfusion; Evans dye perfusion

2% Evans blue dye (Sigma E2129-10G) was prepared in saline. The dye was used as the initial test for peripheral perfusion within the abdominal wall graft at different time points of MP. Images were taken by camera from the surface of the muscle. Stained muscle provided evidence for peripheral perfusion.

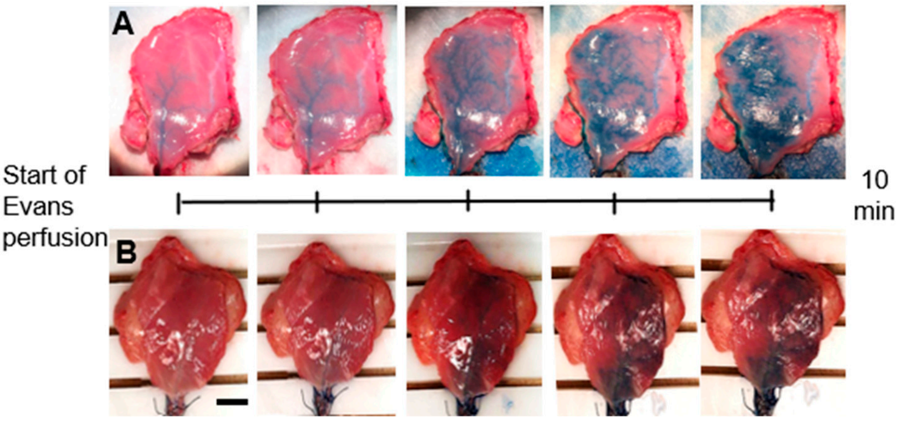

Supplementary Figure S3. Intubation of deep inferior epigastric in abdominal wall graft provides viable access for MP. Representative images (taken every 2 mins) of Evans blue dye perfusion into abdominal wall graft **A,** immediately after procurement surgery **B,** after 12-hour perfusion with Perfadex. The scale bar is 1 cm.

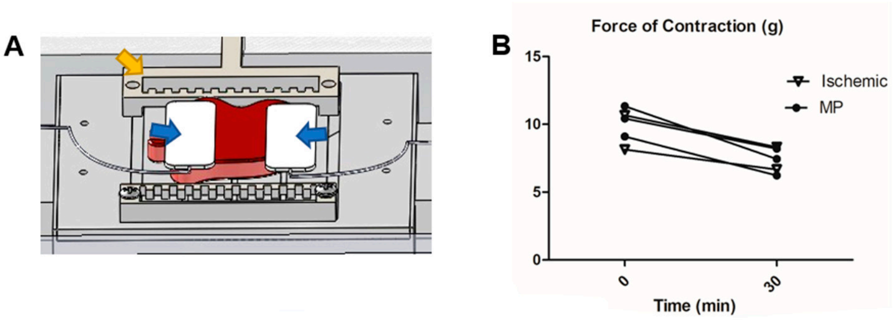

Supplementary Figure S4. Direct electrical stimulation in the bioreactor can deliver biphasic signals and record the force of abdominal wall muscle contraction. A. Illustration of abdominal wall graft mounted in the bioreactor covered by surface electrodes (yellow arrow shows force sensor clamp, blue arrows show electrodes). **B.** Force

sensor reading of abdominal wall muscle contraction over 30 minutes with and without MP. All the grafts had been ischemic for 30-40 minutes prior to time point 0 (n=3).

<u>Oxygenator characterization</u>

Oxygen transfer in the composite membranes with the recirculating flow was evaluated by monitoring dissolved oxygen versus time in the reservoir. Assuming a complete mixed liquid phase, which is reasonable with recirculation, the reservoir concentration vs. time data was analyzed using the following equation[42]. Volumetric mass transfer coefficient (*kLa*) in the liquid medium is deduced from the slope of the curve described by:

$$\frac{dC}{dt} = \mathrm{k_L a}\,(\mathrm{C_e} - \mathrm{C})$$

$$\int_{\mathrm{C_1}}^{\mathrm{C_2}} \frac{1}{(\mathrm{C_e} - \mathrm{C})}\,\mathrm{dC} = \mathrm{k_L a} \int_{\mathrm{t_1}}^{\mathrm{t_2}} \mathrm{dt}$$

$$\ln\left(\frac{\mathrm{C_e} - \mathrm{C_1}}{\mathrm{C_e} - \mathrm{C_2}}\right) = \mathrm{k_L a t}$$

The duration of one experiment (*i.e.* one *kLa* measurement) was greater than 30 minutes and was thus negligible when compared to the response time of the probe (0.5 s) [43]. The interfacial area offered to mass transfer (*a*) is a membrane porous surface $0.2 m^2$

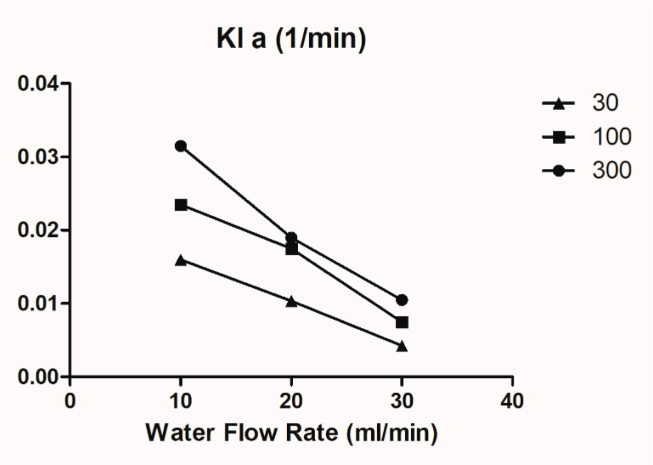

Supplementary Figure S5. Volumetric mass transfer coefficient in relation to perfusate (water) flow rate and gas (oxygen) pressure.

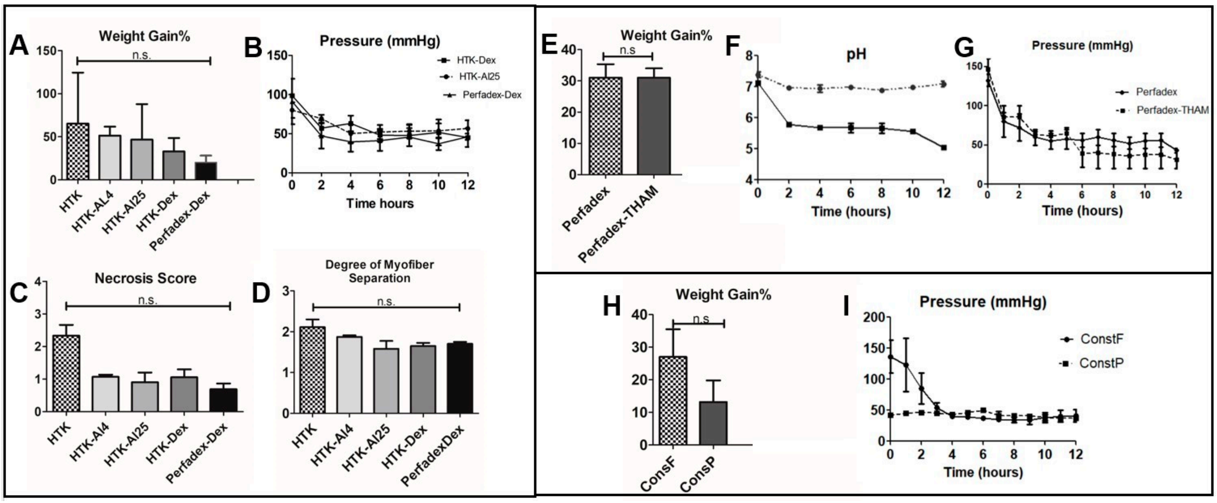

Supplementary Figure S6. Determining perfusate composition and perfusion protocol for sub-normothermic MP of rat abdominal wall grafts. (**A-D**) Comparisons of Perfadex versus HTK, HTK+4% albumin, HTK+25% albumin, and HTK + dextran to study osmotic pressure perfusion-induced edema**. A.** Weight gain after 12-hour MP. **B.** Necrosis score after 12-hour MP. **C.** Recorded perfusion pressure. **D.** Degree of myofiber separation in perfused samples. **(E-G)** Comparisons of Perfadex versus Perfadex + THAM buffer to study osmotic pressure perfusion-induced edema. **E.** Weight gain after 12-hour MP with Perfadex supplemented w/wo THAM. **F.** Outflow perfusate pH versus time. **G.** Recorded perfusion pressure. **(H-I)** Comparisons of constant-flow (0.2 ml/min) versus constant-pressure (45mmHg) MP with Perfadex + THAM buffer to study osmotic pressure perfusion-induced edema. **H.** Weight gain after 12-hour of perfusion. **I.** Recorded perfusion pressure. (n=3 per group)

Perfusate reservoir

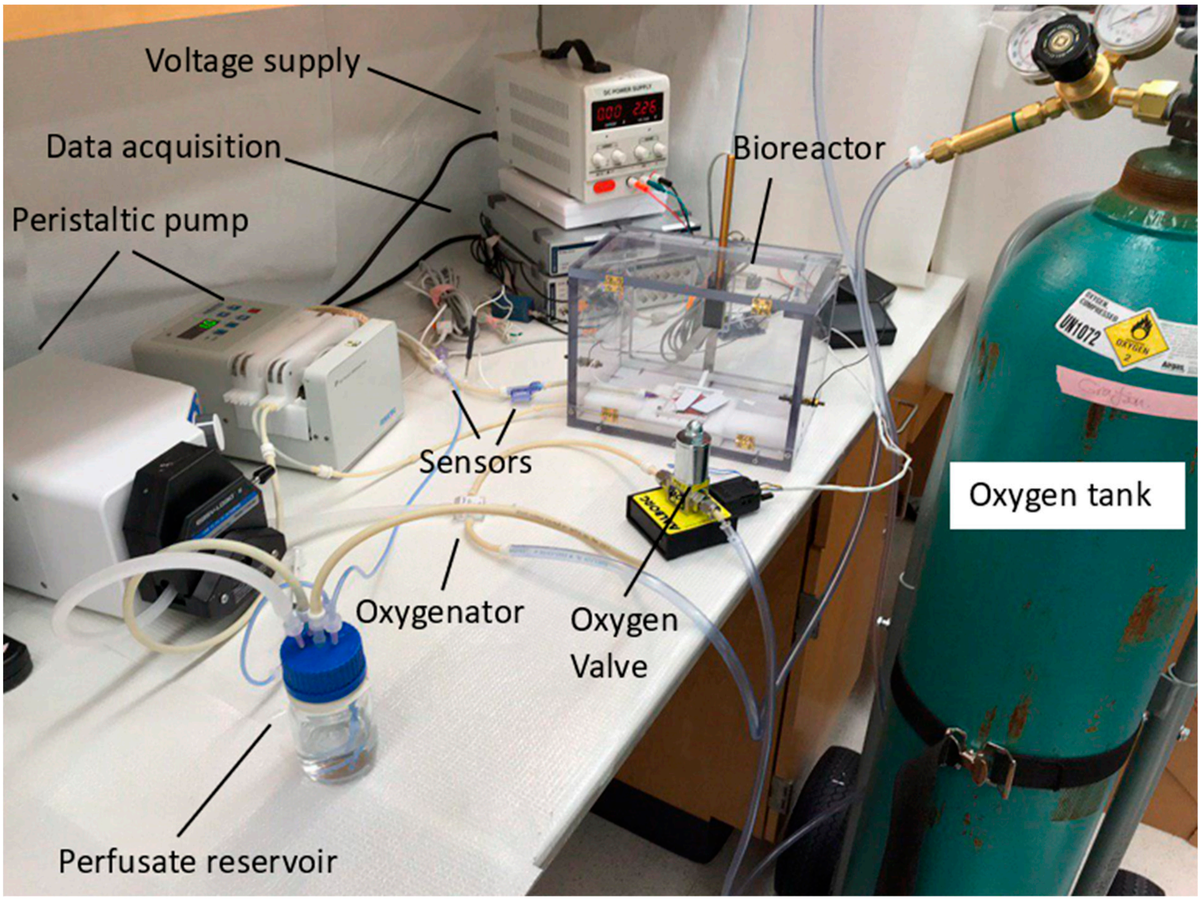

Supplementary Figure S7. Image of the MP system configured to preserve an abdominal wall VCA. The first peristaltic pump transported the perfusate continuously from the reservoir to the bioreactor. At the same time the second peristaltic pump recirculated the perfusate between the oxygenator and reservoir. The solenoid valve controlled oxygen flow through the oxygenator. Multiple sensors were implemented in tubing to monitor perfusion in real-time.
